# Glial Fibrillary Acidic Protein (GFAP) as a Mesenchymal marker of Early Hepatic Stellate Cells Activation in Liver Fibrosis in Chronic Hepatitis C Infection

**DOI:** 10.12669/pjms.305.5534

**Published:** 2014

**Authors:** Sobia Hassan, Serajuddaula Syed, Shahnaz Imdad Kehar

**Affiliations:** 1 Dr. Sobia Hassan, Lecturer, Pathology Department, Ziauddin University Clifton Campus, Karachi, Pakistan.; 2 Prof. Serajuddaula Syed, Head of Pathology Department, Ziauddin University Clifton Campus, Karachi, Pakistan.; 3 Dr. Shahnaz Imdad Kehar, Associate Professor, Pathology Department, BMSI – JPMC, Karachi, Pakistan.

**Keywords:** Glial Fibrillary Acidic Protein (GFAP), Alpha Smooth Muscle Actin (α-SMA), Liver Fibrosis, Hepatic Stellate Cells (HSCs)

## Abstract

***Objective:*** This study aims to determine expression of Glial Fibrillary Acidic Protein and of Alpha Smooth Muscle Actin (α-SMA) in hepatic stellate cells of CHC cases and their association with stage of fibrosis.

***Methods:*** The study was conducted at Ziauddin University, Clifton Campus during the **year 2010-2012.** Sixty Chronic Hepatitis C cases were immmunostained using anti α-SMA antibody and anti-GFAP antibody. Semi quantitative scoring in pericentral, periportal and perisinusoidal area of each case was done to assess immunoexpression of each marker.

***Results*** *:* Immunoexpression of GFAP showed significant association with α-SMA. GFAP expression was inversely correlated with progression of fibrosis.

***Conclusion*** *:* GFAP could represent a useful marker for early hepatic stellate cells activation. Follow up biopsies showing decline in GFAP levels may help identify the target group requiring aggressive therapy.

## INTRODUCTION

World Health Organization (WHO) has assessed that about 170 million people in the world are suffering from with Hepatitis C Virus (HCV), more than half of which advance to chronic liver disease.^1^ By the end of 2015, a fourfold escalation in the prevalence of chronic hepatitis C has been anticipated by Center for Disease Control (CDC).^2^

The main damage caused by HCV is hepatic fibrosis. The Hepatic Stellate Cells (HSCs) activity reflects the chief event in hepatic fibrogenesis.^3^ HSCs are a resident of perisinusoidal space (or space of Disse) which is between the endothelial wall of the sinusoid and the vascular surface of the hepatocytes.^4^ Due to the cytokines produced by injured hepatocytes, HSCs lose their retinols and are converted into myofibroblasts which are contractile and fibrogenic.^5^ These transformed HSCs express some mesenchymal markers including Alpha Smooth Muscle Actin (α-SMA), which is a reliable and widely used marker of activated HSCs.^5^^,^^6^

Glial Fibrillary Acidic Protein (GFAP), first categorized in astroglial cells, is a member of intermediate filaments which maintains cell’s mechanical strength and structure.^7^ Hepatic expression of GFAP has been reported at diverse stages of human chronic hepatitis. It has been documented that GFAP could represent a more useful marker of early HSCs activation than α-SMA.^8^ To our knowledge no such study has been done/ published in Pakistan.

The aim of this study was to appraise the structural characteristics and distribution of HSCs expressing both GFAP and SMA in chronic hepatitis C and to associate these markers with stages of fibrosis and necroinflammatory grades in CHC patients.

## METHODS

This cross sectional study was carried out on liver biopsy of 60 separate chronic hepatitis C patients, collected during 2010-2012. The study was approved by the ethical review committee of Ziauddin University. The biopsies were taken from the archives of Institute of Basic Medical Sciences, Jinnah Postgraduate Medical Centre, Karachi and The Laboratory, Saddar, Karachi. The study was conducted at pathology laboratory, Ziauddin University, Clifton campus and immunostaining was performed at BMSI - JPMC. Blocks of formalin fixed, paraffin embedded liver biopsy of PCR proven chronic hepatitis C patients were taken. Serial sections of 5μm were cut from the paraffin blocks. The histopathology and immunohistochemistry were then performed.

For Histopathology, routine Hematoxylin and Eosin staining was done and liver fibrosis was evaluated using Metavir scale.^9^ Every specimen was staged for fibrosis on a five-point scale; F0 = no fibrosis; F1 = portal fibrosis without septa; F2 = portal fibrosis with rare septa; F3 = numerous septae without cirrhosis; and F4 = cirrhosis. The activity, which is the amount of necroinflammation, is graded on a 4-point scale from A0 to A3. A0 = no histological activity, A1 = mild activity, A2 = moderate activity, and A3 = severe activity. Score of less than F3 and A2 was taken as low score; a score of F3 and above & A2 and above was taken as high score.^10^^,^^11^

For immunohistochemistry, sections were mounted on glass slides coated with poly- L- lysine. After de-paraffinization, quenching was done using H_2_O_2_. Antigen retrieval was done by using EDTA in a preheated water bath for 20 minutes. Duplicate liver sections were incubated with primary antibody i.e. ready-to-use mouse monoclonal anti α-SMA (Cell Marquee, USA) for 30 minutes and ready-to-use mouse monoclonal anti GFAP (Cell Marquee, USA) for one hour (according to manufacturer’s instructions). Positive and negative control slides were included within each session. After washing with PBS, sections were incubated for 20 minutes in secondary antibody (HRP), followed by 20 minute incubation in tertiary antibody (HRP plus). The reaction was visualized using diaminobenzidine followed by counterstaining with Hematoxylin. The immunoexpression of both α-SMA and GFAP on HSCs was scored separately in periportal, pericentral and perisinusoidal areas. The total number of HSCs immunostained by α-SMA and GFAP was determined semi quantitatively as 0: no staining or less than 3% of the region; I: positive for 3- 33% of the region; II: positive for 34-66% of the region; and III: positive for more than 66% of the region.^8^^,^^11^

***Statistical Analysis*** *: *Statistical software SPSS version 20.0 was used for data feeding and analysis. For quantitative variables mean with standard deviation was calculated. For qualitative/ categorical variables percentages and frequencies were calculated. Chi square was used to show association between GFAP and α-SMA on HSCs. In all statistical analysis, only p-value <0.05 was considered to be significant.

## RESULTS

Formalin fixed, paraffin embedded blocks of sixty separate patients suffering from chronic hepatitis C were taken. There were 52% males and 48% females. Their ages ranged from 19 years to 53 years with mean+SD of 37.4+8.4. The total number of HSCs immunostained by α-SMA and GFAP was determined semi quantitatively. HSCs were recognized as stretched cells with long cytoplasmic processes and central nucleus (Fig. 1 & 2).

The α-SMA positive HSCs were found to be mainly located in perisinusoidal region (Table I & II). It was found that α-SMA positive HSCs were detectable in all stages of fibrosis. However, the immunoexpression in lower stage of fibrosis (F≤ 2) was significantly higher than the immunoexpression in high stages (p=0.001). Likewise, in patients with low necroinflammatory grade (A0-A1) showed higher immunoexpression when compared to patients with higher necroinflammatory grade (p=0.025). In contrast the periportal and pericentral areas did not show statistically significant changes with grades and stages of CHC (Table I & II).

Like α-SMA, the GFAP positive cells were also seen to be more prominent in perisinusoidal area as compared to periportal and pericentral areas. For low fibrosis stages, the expression of GFAP on perisinusoidal HSCs was the highest, with a significant difference with higher stages (p=0.001). The Immunoexpression of GFAP positive HSCs showed that in cases with low grade necroinflammatory activity (A0-A1) a significantly higher expression of GFAP-positive HSCs was detected in the perisinusoidal zone when compared with higher grades of activity (A2-A3)(p=0.001). The periportal HSCs also showed higher expression in low necroinflammatory grade (p=0.023). However, there was no significant difference in fibrosis stages in the periportal area. The pericentral area did not show significant changes in various stages and grades of CHC.

The results showed that in perisinusoidal area, the overall frequency of immunoexpression of GFAP (78.4%) is higher than that of SMA (2.7%). (Table-II)

Among the studied HCV-infected patients, there was a significant association between the expression of α-SMA and GFAP in all areas: perisinusoidal, periportal and pericentral areas (p=< 0.001) (Table-III).

## DISCUSSION

Hepatic fibrosis is the basic damage resulting from CHC, which is amongst principal health problems.^12^ Hepatic fibrosis is a wound healing process which results from accunulation of extracellular matrix (ECM) eventually leading to architectural modifications in the liver parenchyma.^13^ Liver Fibrosis may not present clinically until an advanced or cirrhotic stage.^14^ Liver biopsy has been the foundation for diagnosis of hepatic pathologies and denotes the gold standard for assessment of hepatic fibrosis. The liver biopsy is usually performed under sonographic guidance as an outpatient technique, which paralleled to blind Percutaneous Liver Biopsy (PLB), is not only cost effective, but also curtails the potential perils related to blind PLB.^15^ Moreover, biopsy offers supplementary facts about any unrecognized hepatic disease.^16^

**Table-I:** Immunoexpression of GFAP and α-SMA on HSCs in CHC Patients with Necroinflammatory activity (n=60).

Immunoexpression		Necroinflammatory activity		p-value
		Low grade (0-1) (n=22)	High grade (2-3) (n=38)	
		%	%	
GFAP on HSCs				
Perisinusoidal	1	0.0	42.1	0.001 **
	2	4.5	36.8	
	3	95.5	21.1	
	
Periportal	0	0.0	7.9	0.023 *
	1	50.0	73.7	
	2	50.0	18.4	
	
Pericentral	0	36.4	39.5	0.270
	1	45.5	55.3	
	2	18.2	5.3	
α-SMA on HSCs				
Perisinusoidal	0	0.0	2.6	0.025 *
	1	9.1	42.1	
	2	86.4	55.3	
	3	4.5	0.0	
	
Periportal	0	0.0	5.3	0.476
	1	54.5	47.4	
	2	45.5	42.1	
	3	0.0	5.3	
	
Pericentral	0	27.3	28.9	0.735
	1	54.5	50.0	
	2	18.2	15.8	
	3	0.0	5.3	

* Significant p<0.05,

** highly significant p<0.01

**Table-II:** Immunoexpression of GFAP and α-SMA on HSCs in CHC Patients with Stage of fibrosis (n=60).

Immunoexpression		Stage of fibrosis		p-value
		Low stage (0-2) (n=37)	High stage (3-4) (n=23)	
		%	%	
GFAP on HSCs				
Perisinusoidal	1	0.0	69.6	0.001 **
	2	21.6	30.4	
	3	78.4	0.0	
	
Periportal	0	0.0	13.0	0.059
	1	64.9	65.2	
	2	35.1	21.7	
	
Pericentral	0	40.5	34.8	0.837
	1	48.6	56.5	
	2	10.8	8.7	
α-SMA on HSCs				
Perisinusoidal	0	2.7	0.0	0.001 **
	1	10.8	60.9	
	2	83.8	39.1	
	3	2.7	0.0	
	
Periportal	0	2.7	4.3	0.053
	1	62.2	30.4	
	2	35.1	56.5	
	3	0.0	8.7	
	
Pericentral	0	32.4	21.7	0.215
	1	54.1	47.8	
	2	13.5	21.7	
	3	0.0	8.7	

** Highly significant p<0.01

**Table-III:** Association of Immunoexpression of GFAP (Perisinusoidal) and α-SMA (Perisinusoidal) on HSCs

Immuno-expression		α-SMA (Perisinusoidal) on HSCs				Total
		0	1	2	3	
		%	%	%	%	%
GFAP (Perisinusoidal) On HSCs	1	0	81.3	18.8	0	100
	2	0	20	80	0	100
	3	3.4	6.9	86.2	3.4	100
Total		1.7	30	66.7	1.7	100

Significant Association of Immunoexpression of GFAP (Perisinusoidal)

and α-SMA (Perisinusoidal) on HSCs (p<0.01).

**Fig.1a:**
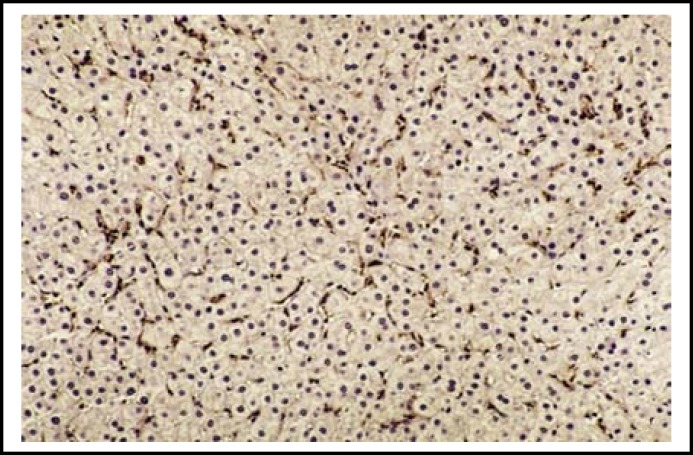
Perisinusoidal α-SMA-positive HSCs in a liver section with CHC with high METAVIR score (A2F3). (Immunohistochemistry x 200)

**Fig.1b:**
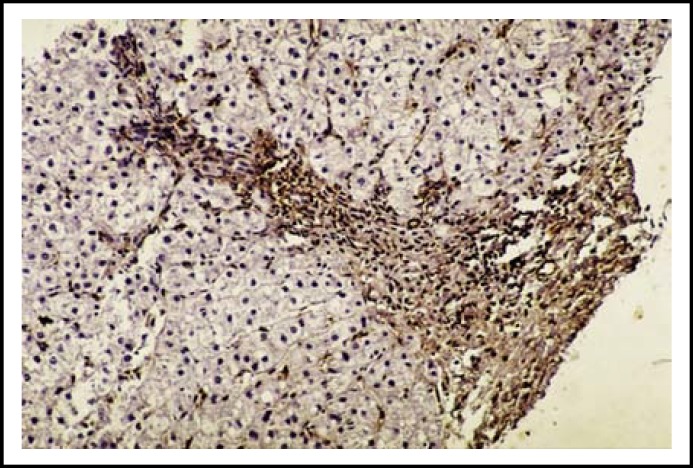
Periportal α-SMA-positive HSCs in a liver section with CHC of high METAVIR score (A2F3). (Immunohistochemistry x200)

**Fig.2a:**
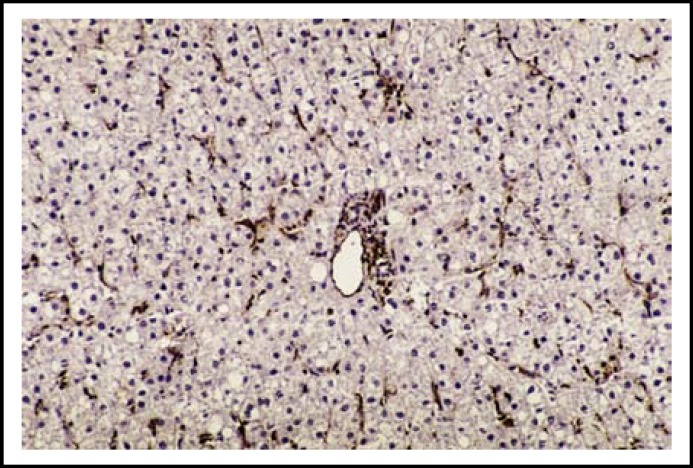
Many perisinusoidal GFAP-positive HSCs within the hepatic parenchyma in a liver section of CHC with low METAVIR score (A1F1). (Immunohistochemistry x 200)

**Fig.2b:**
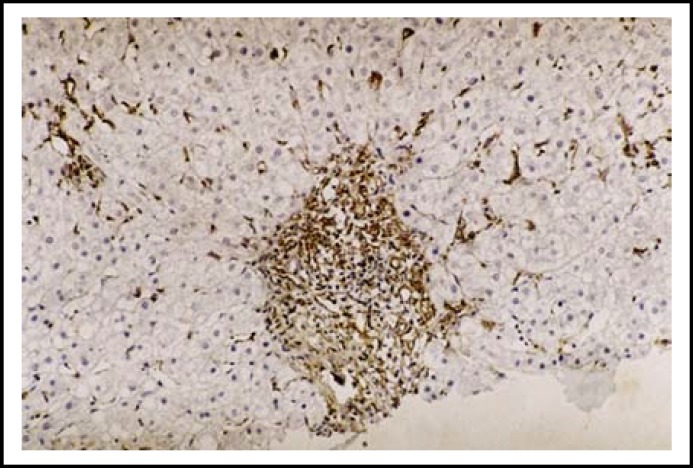
Periportal area showing GFAP positive cells in liver section of CHC with low Metavir score (A1F1). (Immunohistochemistry x 400)

The Hepatic Stellate Cells are the main contributors of hepatic fibrosis and HSCs activity has been quantified with reference to the magnitude of fibrosis & necroinflammatory activity.^4^,^17^ The activated stellate cells express certain mesenchymal markers including α-SMA.^18^,^19^ The α-SMA expression is strikingly augmented in CHC due to stellate cells activation. Hence it has been shown to be a useful marker for detecting hepatic fibrosis.^19^ However, the association between the α-SMA-positive HSCs and the magnitude of fibrosis is debatable. In this study the immunoexpression of α-SMA positive cells was seen to be more prominent in perisinusoidal areas.^11^ This may be due to HSCs being in apposition with sinusoids, thereby responding to early endothelial modifications after injury. The α-SMA positive HSCs were found to be significantly detectable in all stages and grades of CHC.^11^ It was observed that immunoexpression was more significant in lower stage of fibrosis (F≤ 2) than in high stage.^8^, ^11^ This may be because they were already activated by the virus infection, even in the absence of marked fibrosis. Likewise, the expression of α-SMA is higher in lower necroinflammatory grades as compared to the higher grades of necroinflammation. This observation is consistent with the previous studies.^8^^,^^11^

Glial Fibrillary Acidic Protein expression was initially described in resting stellate cells in vivo, and it was shown that there was a high expression in the acute response to injury in the rat, while in the chronic injury low expression was shown.20 In the present study, it was observed that the immunoexpression of GFAP positive cells was also more prominent in perisinusoidal areas like α-SMA.8 This was also because of the close proximity of HSCs with sinusoids. Similar finding has been reported in nervous system where injury is associated with increased GFAP expression in astrocytes positioned close to blood brain barrier.21 In perisinusoidal area, we observed that low necroinflammatory grade (A0-A1) showed a significantly higher expression of GFAP-positive HSCs when compared with high necroinflammatory (A2-A3). Similar observation was made with fibrosis stage where low stages showed highest immunoexpression of GFAP in perisinusoidal HSCs , with a significant difference with higher stages .Unlike, α-SMA the patients with stages F3-F4 did not show strong immunostaining with GFAP in perisinusoidal HSCs. The GFAP positive stellate cells may be antecedents of the HSCs detected by α-SMA immunostaining or they may denote a diverse subpopulation. ^8^

The periportal HSCs also showed higher GFAP expression in low necroinflammatory grade but significant difference in fibrosis stages could not be established. The pericentral area did not show significant changes in different stages and grades of CHC.

The α-SMA is a well-known and reliable mesenchymal marker of HSCs activation.^8^^,^^18^ Our observation of strong association of GFAP with the gold standard immunohistochemical marker, α-SMA, suggests that GFAP could be a useful indicator of early HSCs activation in CHC patients.

A limitation of our study was smaller number of cases so we could not authenticate our results in a different cohort of patients. Also, normal healthy liver biopsies were not available to be used for comparison.

## CONCLUSION

Our study shows that GFAP can be considered as a useful marker for diagnosis of early hepatic fibrosis in CHC patients. The use of GFAP may help treat CHC patients at a stage when fibrotic changes are mild because once end-stage cirrhosis establishes, liver transplantation is the sole modality of treatment. Also, GFAP may be used for follow-up of patients where a significant decrease in GFAP expression on HSCs, even without abnormal histopathological changes in grading or staging, can be considered for target therapy. 

## Notes

***Funding: ***Self-funded.
